# Withdrawal: MicroRNA-7 compromises p53-dependent apoptosis by controlling the expression of the chromatin remodeling factor SMARCD1

**DOI:** 10.1074/jbc.W120.016987

**Published:** 2021-01-13

**Authors:** Chun-Fu Hong, Shu-Yu Lin, Yu-Ting Chou, Cheng-Wen Wu

VOLUME 291 (2015) PAGES 1877–1889

This article has been withdrawn by all the authors. The Journal pointed out that the p53 immunoblot from H1975 in Fig. 3*F* was reused in Fig. 4*E* (α-tubulin) and Fig. 5*A* (p53 from A549), representing different experimental conditions. There are duplicated immunoblots representing different experimental conditions in the last column of Fig. 4*B* (α-tubulin) and first column of Fig 5*E*. There is a duplicated FACS plot in Fig. 3*G* (H1975 scramble) and Fig. 4*F* (H1975 ctrl). There is a duplicated FACS plot in Fig. 5*D* (H1975 scramble) and Fig. 5*F* (ctrl). There are some duplicated immunoblots in Fig. 2*A* (A549 SMARDC1) and Fig. 4*A* (SMARDC1 column 1) as well as duplications with their respective α-tubulin loading controls. The authors state that the errors occurred during preparation of the figures and figure legends and state that all of the results reported in this article are valid. Through a full discussion, all authors agree on the decision to withdraw the paper.

